# On the basis set convergence of electron–electron entanglement measures: helium-like systems

**DOI:** 10.3389/fchem.2013.00024

**Published:** 2013-11-01

**Authors:** Thomas S. Hofer

**Affiliations:** Theoretical Chemistry Division, Institute of General, Inorganic and Theoretical Chemistry, University of InnsbruckInnsbruck, Austria

**Keywords:** electron correlation, electron electron entanglement, entanglement entropy, helium-like systems, basis set convergence

## Abstract

A systematic investigation of three different electron–electron entanglement measures, namely the von Neumann, the linear and the occupation number entropy at full configuration interaction level has been performed for the four helium-like systems hydride, helium, Li^+^ and Be^2+^ using a large number of different basis sets. The convergence behavior of the resulting energies and entropies revealed that the latter do in general not show the expected strictly monotonic increase upon increase of the one–electron basis. Overall, the three different entanglement measures show good agreement among each other, the largest deviations being observed for small basis sets. The data clearly demonstrates that it is important to consider the nature of the chemical system when investigating entanglement phenomena in the framework of Gaussian type basis sets: while in case of hydride the use of augmentation functions is crucial, the application of core functions greatly improves the accuracy in case of cationic systems such as Li^+^ and Be^2+^. In addition, numerical derivatives of the entanglement measures with respect to the nucleic charge have been determined, which proved to be a very sensitive probe of the convergence leading to qualitatively wrong results (i.e., the wrong sign) if too small basis sets are used.

## 1. Introduction

While the foundations of quantum theory were laid out already at the beginning of the 20th century (Planck, 1901; Einstein, 1905), their influence on chemical science become only apparent after the influential work of Schrödinger in the 1920's (Schrödinger, 1926a,b,c,d), correctly predicting the non-relativistic energy eigenvalues for hydrogen-like systems from first principles. Schrödinger's formulation of the quantization as an eigenvalue problem of a wave equation essentially marked the starting point of modern electronic structure theory aimed at the quantum mechanical description of many-electron systems (Szabo and Ostlund, 1996; Levine, 1999; Helgaker et al., 2000; Cook, 2005).

The vast majority of approaches operate within the framework of a number of approximations being the treatment of stationary systems (time–independence), neglect of effects resulting from special relativity (Dyall, 2007) and neglect of nuclear quantum effects (Born and Oppenheimer, 1927). Furthermore, in the simplest case the *n*_*e*_-electron probability function is approximated via a Slater determinant (Slater, 1929), which corresponds to the antisymmetric superposition of all possible products of *n*_*e*_ one-electron functions |ψ_*i*_〉

(1) $$\Psi =\frac{1}{\sqrt{n_{e}!}}\left|\begin{array}{ccccc} \psi_{1}(r_{1}) & \psi_{2}(r_{1}) & \psi_{3}(r_{1}) & \ldots & \psi_{n_{e}}(r_{1})\\ \psi_{1}(r_{2}) & \psi_{2}(r_{2}) & \ldots & \ldots & \psi_{n_{e}}(r_{2})\\ \ldots & \ldots & \ldots & \ldots & \ldots \\ \psi_{1}(r_{n_{e}}) & \psi_{2}(r_{n_{e}}) & \psi_{3}(r_{n_{e}}) & \ldots & \psi_{n_{e}}(r_{n_{e}}) \end{array}\right|$$

with **r**_1_, **r**_2_, …, **r**_*n*__*e*_ being the coordinates of the *n*_*e*_ electrons. The one–electron functions |ψ_*i*_〉 are known as molecular or spin orbitals and are generated via linear combination (Lennard-Jones, 1929) of primitive functions referred to as atomic orbitals.

Application of the corresponding electronic Hamiltonian on the Slater determinant leads to the formulation of the Hartree-Fock (HF) method (Hartree, 1928a,b; Fock, 1930), in which a numerical solution of Schrödinger's equation is obtained via a variational principle: by optimizing the coefficients used in the linear combination of atomic orbitals in an iterative way, the lowest energy and hence, the best approximation to the wave function is obtained.

Although the HF energy is in many cases close to the exact non-relativistic energy (>99%), the error resulting from the use of a product ansatz may lead to dramatic deviations of observables derived from from the Hartree-Fock wave function. The reason of this short-coming is the fact that in the HF approach electrons interact with each other only via their mean fields, i.e., the motion of the electron is uncorrelated and the energy difference between the Hartree-Fock result and the exact, non-relativistic energy of the system (formally calculated using a complete one–electron basis) is known as correlation energy.

During the last decades research in theoretical chemistry focused extensively to overcome this limitations and impressive progress was achieved in formulating and optimizing a variety of so-called post-HF methods (Szabo and Ostlund, 1996; Levine, 1999; Helgaker et al., 2000; Cook, 2005). Instead of using a single Slater determinant these methods employ a number of determinants to formulate the wave function. The exact solution to the Schrödinger equation in a given one–electron basis can be obtained via a linear combination of all possible Slater determinants (Helgaker et al., 2000), but due to the enormous number of determinants the application of this approach known as Full Configuration Interaction (FCI) is still limited to the smallest systems.

At this point it should be noted that in these approaches the treatment of correlation effects rests solely in the responsibility of the wave function, whereas the Hamiltonian of the system remains unmodified. The fact that a “correlation operator” cannot be defined implies that electron correlation is a purely methodical effect resulting from the use of an inadequate trial wave function in the Hartree-Fock approach. Consequently, the phenomenon of electron correlation is widely believed to have no resemblance in the physical world.

However, during the last two decades a different view of correlation has emerged in the context of quantum information theory (Nielsen and Chuang, 2010; Fayngold and Fayngold, 2013) by considering electron correlation as an entanglement phenomenon (dinger, [Bibr B49]). Following Collins' conjecture (Collins, 1993) the correlation energy of a system is proportional to the respective entropy of entanglement, typically expressed via the von Neumann entropy or related measures. It can be shown (Ghirardi and Marinatto, 2004; Kais, 2007; Wang and Kais, 2007) that a wave function composed of a single Slater determinant does not violate Bell's inequality, implying that the Hartree-Fock ansatz treats a disentangled state, whereas the wave function used in post-HF methods such as FCI accounts for entanglement effects.

The route to investigate correlation phenomena via entanglement measures of model systems enabling analytic solutions of Schrödinger's equation [such as the Crandall and Hooke atoms (Manzano et al., 2010)] or real atomic species such as helium-like systems (Huang and Kais, 2005; Manzano et al., 2010; Dehesa et al., 2012; Benenti et al., 2013) treated with post-HF methods has already attracted the interest of a number of researchers. In the latter case either very accurate one-electron basis functions of the Kinoshita type or small Gaussian or Slater type orbital (GTO, STO) basis sets have been employed (Huang and Kais, 2005; Benenti et al., 2013). In the first case very accurate estimations of energy and entanglement entropy are possible, however it is difficult to extend the approach to general systems. The use of a GTO basis on the other hand is widely used in modern electronic structure theory, however the Gaussian nature inherent to this type of basis is known to limit the achievable accuracy. Furthermore, despite the availability of high quality basis sets in the literature, only small one-electron bases (e.g., 3-21G, 6-31G and cc-pV5Z) have been used in the past to investigate entanglement phenomena in helium like systems.

Further systems used to investigate entanglement and its relation to electron correlation are the hydrogen molecule (Gersdorf et al., 1997; Huang and Kais, 2005; Wang and Kais, 2007; Esquivel et al., 2011; Vesaghi et al., 2011) as well as other many-electron (*n*_*e*_ > 2) systems such as the unifrom electron gas (Ziesche, 1995), atom-like species (Esquivel et al., 1996; Sagar et al., 2002) and small molecular compounds (Ramírez et al., 1997; Maiolo et al., 2007; Esquivel et al., 2011). In all cases it was shown that the entanglement measures show similar trends compared to the correlation energy, but as in the case of helium-like systems only small GTO-type basis sets were considered in these investigation.

Despite the rather small one–electron bases employed in previous studies, these works clearly demonstrate the relation between correlation and entanglement providing numerical evidence confirming Collin's conjecture. It is, thus, of considerable interest to investigate this dependence upon an increase of the quality of the one-electron basis. While the energy of the system is known to decrease monotonically toward the exact, non-relativistic energy, it is not obvious whether a similar tendency, namely a strictly monotonic increase, is observed in case of the entanglement measures. The latter would be required for a direct correlation of these two properties as implied by Collin's conjecture. Data provided by Ramírez et al. (1997) using eight different Pople–type basis sets of increasing size indicate, that the monotonic decrease of the correlation energy upon increase of the number of basis functions is accompanied with a monotonic increase of the entanglement entropy. As only small, Pople-type basis sets (highest angular momentum *l* = 2) have been used in this study, which are in general not recommended in computations taking correlation effects into account, a re–investigation of the basis set convergence using high-quality correlation–consistent bases of increasing size (highest angular momentum *l* = 7) appears promising.

Since for helium-like systems the only variable capable of influencing the entanglement is the charge of the nucleus *Z*, the dependence with the respect to the nucleic charge is also of considerable interest. A further question in this context is how well the best GTO type bases predict the entanglement compared to the more accurate Kinoshita type approaches? Shi and Kais have demonstrated that the basis set expansion can be related to finite size scaling and criticality of entropy as the nuclear charge is varied (Shi and Kais, 2004).

The objective of this work was to systematically investigate these questions by comparing different electron entanglement measures for four helium-like systems as a function of the quality of the basis sets as well as the nucleic charge.

## 2. Methodology

In order to compute entanglement properties of the systems envisaged in this study, the wave function |Ψ〉 of the pure two-electron state has to be determined, which can be written as follows

(2) $$|\Psi \rangle =\sum_{a=1}^{2N}\sum_{b=1}^{2N}\omega_{ab}c_{a}^{†}c_{b}^{†}|0\rangle$$

where *c*^†^ correspond to the fermionic creation operators, |0〉 is the respective vacuum state. The indices *a* and *b* run over all 2*N* orthonormal one electron states. Due to antisymmetry the expansion coefficients ω_*ab*_ satisfy ω_*ab*_ = −ω_*ab*_ and ω_*ab*_ = 0 must be fulfilled for *a* = *b*.

The reduced one electron density matrix with respect to electron *A* is obtained via the partial trace of the density matrix over the index of electron *B*:

(3) $$\rho_{A}=Tr_{B}|\Psi \rangle \langle \Psi |$$

The entanglement of the ground state is then given by the von Neumann entropy *S*_*vN*_ using the reduced density matrix ρ_*A*_, which has been shown to be an appropriate measure of the entanglement of two–particle systems (Gittings and Fisher, 2002; Zanardi, 2002):

(4) $$S_{vN}=-Tr\left(\rho_{A}\log_{2}\rho_{A}\right)$$

An alternative measure often employed in literature (Manzano et al., 2010; Dehesa et al., 2012) corresponding to a simplified form of *S*_*vN*_ is the linear entropy *S*_lin_ obtained by retaining just the leading term of the series expression of the logarithm:

(5) $$S_{\text{lin}}=-Tr\left[\rho_{A}\left(\rho_{A}-1\right)\right]=1-Tr\left(\rho_{A}^{2}\right)$$

The linear entropy constitutes a lower bound to *S*_*vN*_ and since it does not require diagonalization of the density matrix, it is often preferred to characterize the mixing of quantum states. It should be noted that the use of *S*_lin_ has no advantage in the current study because ρ_*A*_ already is a diagonal matrix for all considered systems and hence, a diagonalization step is not required.

The expansion coefficients of the CI wave function can be computed using electronic structure programs capable of executing configuration interaction (CI) computations at the appropriate excitation level. In case of two-electron systems configuration interaction using single and double excitations (CISD) already considers all possible excited states and hence, this level corresponds to the FCI expression

(6) $$|\Psi_{\text{CISD}}\rangle =|\Psi_{0}\rangle +\sum_{ia}T_{ia}|\Psi_{i\to a}\rangle +\sum_{\text{ijab}}T_{\text{ijab}}|\Psi_{ij\to ab}\rangle$$

where |Ψ_0_〉, |Ψ_*i*→*a*_〉 and |Ψ_*ij*→__*ab*_〉 are the reference-state (i.e., Hartree-Fock) and the corresponding excited determinants, the respective amplitudes are given as *T*_*ia*_ and *T*_ijab_. Knowledge of the latter enables the evaluation of the one-electron reduced density matrix ρ_*A*_ and the computation of the entanglement measures *S*_*vN*_ and *S*_lin_. The correlation energy is obtained as the difference between the FCI energy and the HF result for the respective reference state (restricted Hartree-Fock).

In addition to the linear and von Neumann entropies derived via density matrices, a third measure for the electron entanglement is used in the literature (Gersdorf et al., 1997; Maiolo et al., 2007; Esquivel et al., 2011), which is based on the occupation numbers of natural spin orbitals obtained from natural bond orbital (NBO) analysis (Glendening et al.,; Reed et al., 1988; Weinhold, 2012). In this post-processing step the canonical orbitals are localized via diagonalizing of localized blocks of the density matrix. Using the resulting occupation numbers *n*_*i*_ the corresponding occupation number entropy *S*_occ_ can be obtained as

(7) $$S_{\text{occ}}=-\sum_{i=1}^{N}\left(\frac{n_{i}}{2}\log_{2}\frac{n_{i}}{2}\right)$$

where the division by two accounts for the double-occupancy of each NBO in case of closed-shell systems (Wang and Kais, 2007). Table 1 lists the occupation numbers obtained for hydride at HF and FCI level using the simple correlation–consistent polarization valence double zeta (cc-pVDZ) basis set. Due to the full occupation of only one orbital in case of Hartree–Fock the occupation number entropy results as zero (no entanglement), whereas in case of the FCI computation a finite value for *S*_occ_ corresponding to an entangled state is obtained. This finding results from the fact that the total occupation is distributed over a number of natural orbitals, which is, however, in stark contrast to the commonly used concept of assigning pairs of electrons to individual orbitals being equivalent to the disentangled Hartree–Fock occupation.

**Table 1:** **Occupation numbers and occupation number entropy *S*_occ_ obtained from an NBO analysis obtained for H^−^ at HF and FCI level using the cc-pVdZ basis set**.

**Orbital**	**HF**	**FCI**
1	2.00000	1.97877
2	0.00000	0.01499
3	0.00000	0.00208
4	0.00000	0.00208
5	0.00000	0.00208
Sum	2.00000	2.00000
*S*_occ_	0.00000	0.02136

Since NBO analyses are readily available in a number of electronic structure packages and the computation of the reduced density matrix is not required, *S*_occ_ provides an alternative approach to characterize entanglement phenomena. In this case the computation of the total density at the FCI level is required, which may become a computationally limiting step when large basis sets are used.

In addition to a systematic investigation of the entanglement measures *S*_*vN*_, *S*_lin_ and *S*_occ_ their dependence with respect to the nucleic charge *Z* of the system is of increased interest, since for helium like systems the nucleic charge is the only external variable influencing the resulting wave functions. Although computer experiments enable non-integer variations of the nucleic charge *Z*, the quality of the result is strongly linked to the employed basis sets. Test computations of helium using basis sets developed for hydrogen and *vice versa* resulted in very poor results for the total energy (data not shown). Thus, any variation of *Z* should be performed only in a region, where the basis set remains valid for the atom in question. Following this line of thought it seemed more appropriate to compute the derivative of the entanglement measures with respect to the nucleic charge via numerical differentiation

(8) $$\frac{\partial S}{\partial Z}{|}_{i}\approx \frac{S_{i\;-2}-8S_{i-1}+8S_{i+1}-S_{i\;+2}}{12\Delta Z}$$

where the subscripted indices denote the increments/decrements in Δ*Z* from the formal nucleic charge of the species corresponding to the point *i*. Since this numerical differentiation requires four data points above or below the formal charge of the systems, five individual QM computations are required to obtain the entanglement entropies and their respective derivatives for each basis set and system considered. In order to ensure that the basis sets remain valid one-electron bases, a very small value for Δ*Z* of 0.0005 a.u. was employed. The derivatives of the entanglement measures were found to be a very sensitive probe of the quality of the computation, yielding qualitatively wrong results (i.e., the wrong sign) when too simple one-electron bases were used.

A variety of basis sets have been considered in this study. In case of H^−^ and He the small Pople-type bases 3-21G (Binkley et al., 1980), 6-31G (Hehre et al., 1972) and 6-311G (Hariharan and Pople, 1973; Krishnan et al., 1980) with and without polarization (Frisch et al., 1984) and diffuse functions (Clark et al., 1983) have been employed since they have been used in the past to investigate entanglement phenomena. However, in case correlated ab initio methods such as CI are to be employed the use of Dunning's correlation– consistent polarization valence *n*-tuple zeta (cc-pVnZ) basis sets (Dunning, 1989; Kendall et al., 1992; Peterson et al., 1994; Woon and Dunning, 1994; van Mourik, et al., 1999) is recommended and consequently, these bases and their augmented variants (aug-cc-pVnZ, d-aug-cc-pVnZ, t-aug-cc-pVnZ representing augmented, double- and triple-augmented variations) constitute the main focus of this study. While in the case of hydrogen *n* ranges from 2 to 6, a range from 2 to 8 is available for helium. Furthermore, a second series of high level basis sets referred to as mcc-pVnZ (*n* = 3 − 8; the letter m indicating modified) developed by Mielke and coworkers (Mielke et al., 1999, 2002, 2005) is available in case of hydrogen. This modified form of basis developed to achieve an improved description of the H + H_2_ reaction barrier, has also been considered in this study along with the corresponding augmented and double-augmented sets. While not explicitly designed for the treatment of atomic hydrogen species, the larger size of this series (*n* = 3 − 8) compared to the conventional cc-PVnZ bases (*n* = 2 − 6) can be expected to yield well-converged data for atomic systems as well. The application of the diffuse augmentation functions is known to have a significant impact on the description of anionic systems and it is expected that this fact is reflected by the different measures of the electron–electron entanglement in the hydride system. In case the number of augmented basis functions is higher than given in the literature, the rule of Dunning and coworkers to construct additional augmentation functions (van Mourik, et al., 1999) (by dividing the square of the exponent of the most diffuse function by the exponent of the second most diffuse function) was employed.

For Li^+^ and Be^2+^ the cc-pVnZ (Dunning, 1989; Kendall et al., 1992; Peterson et al., 1994; Woon and Dunning, 1994) bases only range from *n* = 2 to 5. Although the respective augmented forms were also considered in this study, the application of the cc-pCVnZ and aug-cc-pCVnZ basis sets (Prascher et al., 2011) was considered to be more important for these systems. These bases include further localized functions to improve the description of the electron density close to the nucleus, which is in many cases crucial for an accurate description of positively charged systems.

In order to demonstrate the increase in size of the chosen bases, the composition of the various correlation–consistent basis sets used in this study in terms of the commonly used reference style (Gaussian primitives)/[contracted functions] is listed in the supplementary material (Tables S1, S2).

All QM calculations were performed with the Gaussian09 package (Frisch et al., 2009). In order to eliminate any influence of parameters used in empirical approaches to generate starting orbitals, a so-called “core guess” employing only the core-Hamiltonian matrix was chosen to generate the initial orbitals. To ensure a proper convergence of the wave functions, the convergence criteria were set to 10^−14^ and 10^−10^ for the Hartree-Fock and FCI calculations, respectively. While most of the computations can be conveniently executed on a state-of-the-art notebook (Intel Core i7, 2.9 GHz, 8 GB RAM), the capacities of the MACH high performance computing facility of the Austrian Center for Scientific Computing were required in case of the largest basis sets used in this study. The execution times for the individual computations depend dramatically on the size of the basis set, ranging from a few seconds up to a few days.

## 3. Results

In the following the results for the four systems hydride, helium, Li^+^ and Be^2+^ are discussed. Only a graphical depictions of the respective data are included in the main manuscript, however all data obtained for the energy, the different entanglement measures and their dependence with respect to the nucleic charge for the different one–electron bases have been listed to high precision in the supporting information (Tables S3–S7).

**Figure 1:**
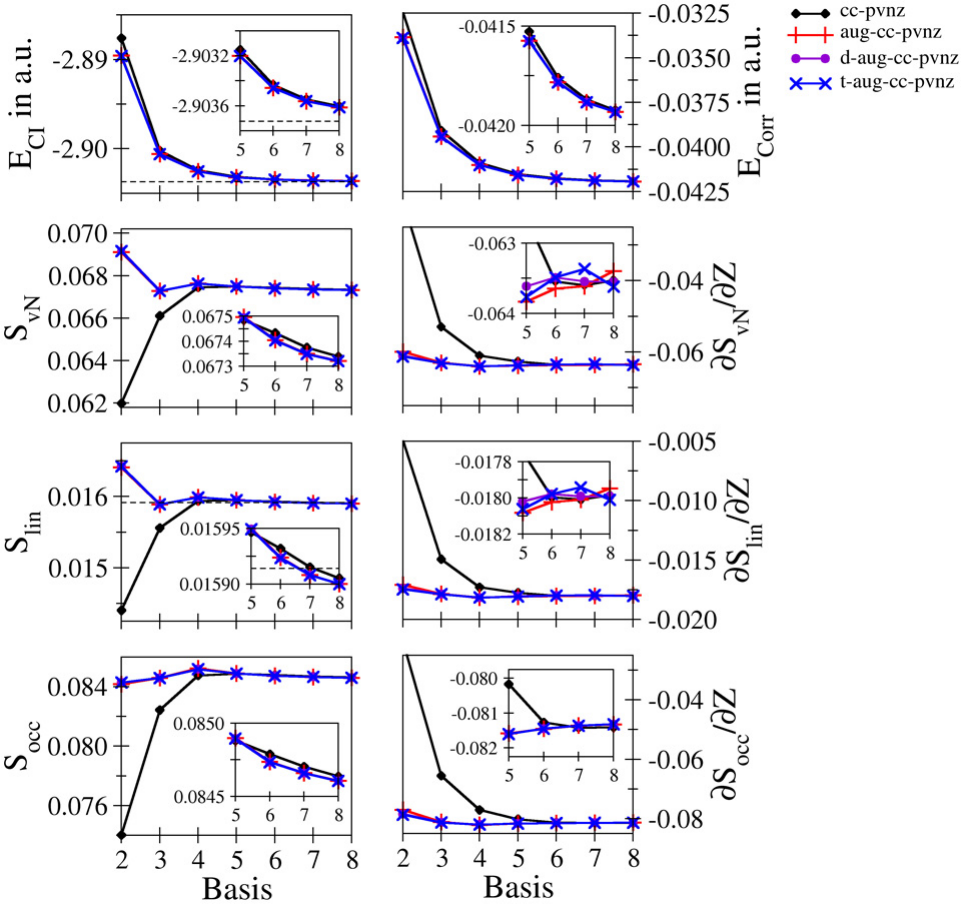
**Total and correlation energy *E*_*CI*_ and *E*_corr_ in atomic units; von Neumann *S*_*vN*_, linear *S*_lin_ and occupation number *S*_occ_ entropies and their derivative with respect to the nucleic charge *Z* obtained for Helium at the FCI level using different basis sets**.

**Helium**: Since the helium atom is the prime example for a real two-electron system, it is considered first in this section. Figure 1 depicts the total and correlation energies *E*_*CI*_ and *E*_corr_, the entanglement measures S_vN_, S_lin_, and S_occ_ and their derivatives with respect to the nucleic charge as a function of the size *n* of the basis set for the cc-pVnZ bases and the respective augmented variants. The respective data along with the values obtained for a number of Pople–type basis sets (3-21G, 6-31G, 6-311G with and without polarization and/or diffuse functions) are given in Table S3. The data agrees well with the scarce number of data available in literature. The values obtained at 3-21 G and cc-pV5Z level agree well with data reported by Huang and Kais (2005) given as 0.0149 and 0.0415 a.u. for *E*_corr_ and as 0.0313 and 0.0675 in case of S_vN_, respectively. (The difference in sign for *E*_corr_ results from a different definition in the calculation of the correlation energy). The entropies compare also well to data obtained using an STO–type basis reported by Benenti et al. (2013) as 0.0785 and 0.01606 for the von Neumann and linear entropies of ground state helium. Although the values differs significantly in case of *S*_*vN*_, *S*_lin_ agrees within 1%.

Both the total as well as the correlation energy show the expected monotonic decrease upon increase of the one electron basis. While the energies obtained from cc-pVnZ bases are found at higher values, the plots of the augmented variants overlap even when magnifying the region *n* = 5 − 8 (see inlay in Figure 1), indicating only a small gain in accuracy when using double- and triple–augmented bases. The total energy converges nicely toward the value reported by (Manzano et al., 2010) using a high–level Kinoshita-type ansatz (dashed line). The deviation between this estimation and the best energy resulting from the largest basis set t-aug-cc-pV8Z is 1.12642 · 10^−4^ a.u., i.e., about one tenth of a milli–Hartree.

The entanglement measures *S*_*vN*_, *S*_lin_ and *S*_occ_ on the other hand show dramatic differences compared to the energies of the system. Foremost, the von Neumann and linear entropies do not show the expected monotonically increasing trend upon increase of the bases: in the cc-pVnZ case an increase is observed in the region *n* = 2 − 5, while the entropies decrease for basis sets beyond cc-pV5Z. In case of all augmented variants of the cc-pVnZ bases, an oscillatory convergence of *S*_*vN*_ and *S*_lin_ is observed, with minima and maxima occurring at *n* = 2 and 3, respectively. Aside from a constant prefactor the von Neumann and linear entropy show a very similar behavior. The values obtained for *S*_lin_ are similar to the estimation given by Dehesa et al. (2012) using a high-level Kinoshita-type description, although it appears that the linear entropy obtained via the GTO bases converges toward a lower limit. Nevertheless, the value for *S*_lin_ resulting from the largest basis set t- aug- cc-pV8Z obtained as 0.015900 is well within the reported range (Dehesa et al., 2012) of 0.015914 ± 4.4 · 10^−5^, the error–limit being the result of a Monte-Carlo numerical integration. Similar as in the case of the energy, the use of additional augmentation functions beyond the first does only lead to minor improvements of the different entanglement measures being visible only on the fifth significant digit.

As expected the increase of the basis led in all cases to a monotonic decrease of the energy, resulting from an improved description of the correlation. Since correlation is associated with entanglement, following Collin's conjecture (Collins, 1993) any entanglement measure should therefore show a monotonic increase, which is, however, not always the case: A decrease of the correlation energy upon increase of the basis is not always linked to an increase of the correlation entropy. It can be seen, however, that the entropies obtained from the different types of the basis sets converge toward the respective basis set limit and hence, it appears that Collins' conjecture holds true in case a complete basis is used, i.e., when employing a large (formally infinite) number of linear independent basis functions.

The convergence of the occupation number entropy *S*_occ_ differs from that of the other two entanglement measures, but it shows an essentially similar behavior in case of the larger basis sets (*n* = 5 − 8). Also in this case the convergence is not monotonic, but shows maxima at *n* = 3 (augmented bases) and *n* = 4 (cc-pVnZ). On the other hand *S*_occ_ is more sensitive with respect to the use of augmentation functions, showing differences already on the third to fourth significant digit.

The numerical derivatives of the entanglement measures with respect to the nucleic charge proved to be more sensitive than the entropies themselves. Although in case of correlation–consistent bases all derivatives are negative, the cc-pVnZ set performs particularly poor, especially for low values of *n*. Even the aug-cc-pVDZ basis shows a notable deviation from the values obtained for the double- and triple augmented bases. While in the case of cc-pVnZ a monotonic decrease of the derivative is observed, the convergence is again oscillatory in case of the augmented variants for all three types of entanglement measures.

The rather bad performance of Pople–type basis sets (see Table S3) with respect to the high-level augmented cc-pVnZ bases is not too surprising, since they have not been developed within the scope of a correlated *ab initio* treatment. However, it should be noted that in case of the simplest basis in this series, the 3-21 G set, the sign of the derivatives of the entanglement measures with respect to *Z* is positive, implying an increase of entanglement upon increase of the nucleic charge. This qualitatively wrong result is observed for all three entanglement measures considered in this study and implies that the use the 3-21 G basis is highly insufficient to properly take correlation/entanglement effects into account. In case the use of Pople–type basis sets is required, a larger veriant such as 6-311 G(2d,2 p) appears to be the minimum choice, leading to similar results for the entanglement entropies than augmented cc-pV4Z bases despite the poor performance in terms of the total energy.

Overall the data suggests that the application of the aug-cc-pV6Z basis appears to be a sufficient compromise between accuracy and computational effort to obtain reasonably converged entanglement measures in case of the helium system.

**Figure 2:**
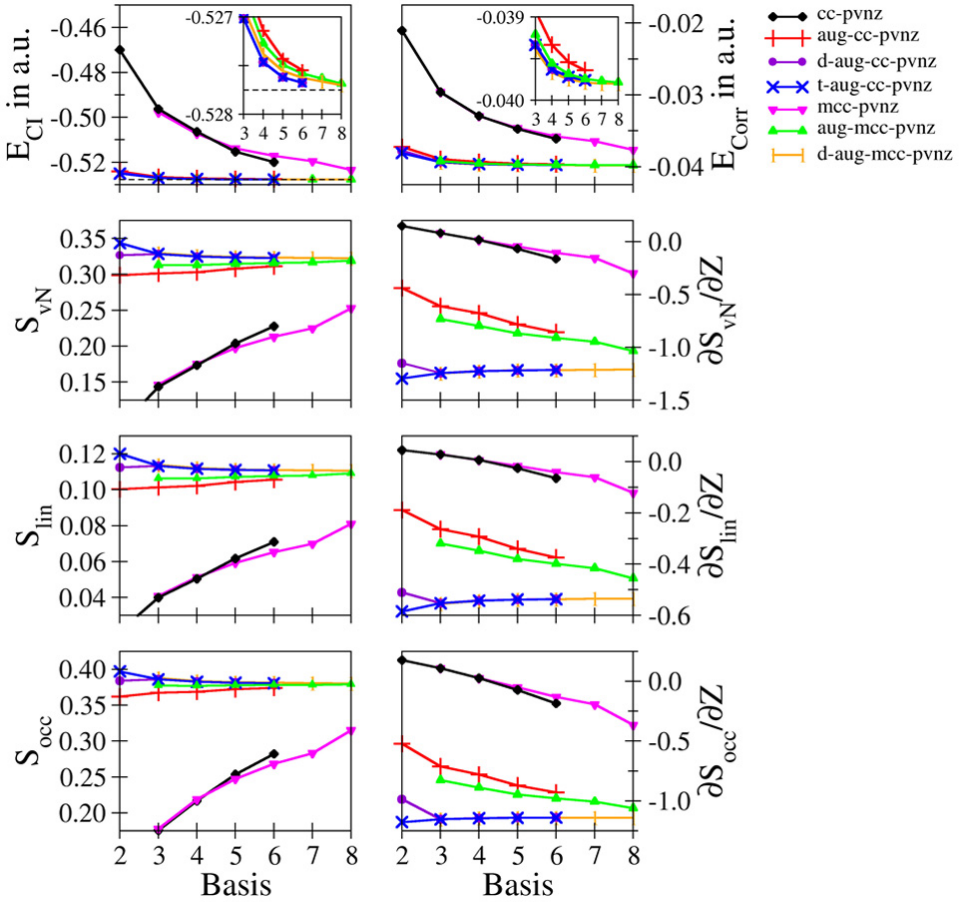
**Total and correlation energy *E*_*CI*_ and *E*_corr_ in atomic units; von Neumann *S*_*vN*_, linear *S*_lin_ and occupation number *S*_occ_ entropies and their derivative with respect to the nucleic charge *Z* obtained for H^−^ at the FCI level using different basis sets**.

**Hydride**: The convergence of the entanglement measures in case of hydride are notably different compared to those of helium. Figure 2 depicts the convergences of the energies, entanglement entropies and their derivatives for the cc-pVnZ and mcc-pVnZ bases as well as the respective augmented sets. The data obtained for various Pople- and Dunning–type basis sets are listed in Table S4, the results for the mcc-pVnZ bases are given in Table S5.

Although the energies *E*_*CI*_ and *E*_corr_ show again a monotonic decrease in all cases as expected, monotonically increasing, monotonically decreasing as well as oscillatory convergence of the entanglement entropies is observed, again being in violation of Collin's conjecture. The energy difference between the largest basis used in this study (d-aug-mcc-pV8Z) and the value reported by Manzano et al. (2010) amounts to 5.15801 · 10^−5^ a.u., (approximately 120th of a milli–Hartree), being on the same order as the energy difference in the helium case.

Due to the absence of diffuse functions in case of the cc-pVnZ and mcc-pVnZ sets, the total as well as the correlation energy show large deviations, which is also reflected by significantly lower values in case of all three employed entanglement measures. Similar as in the helium case treated at the cc-pVnZ level the entropies show a monotonic increase as the description of the system becomes more accurate. However even for the largest sets in these series the entropies are far from converged.

Augmentation of the basis with diffuse functions leads to a significant improvement of energies and entanglement entropies, reflecting the importance of augmentations functions for an accurate quantum chemical treatment of diffuse electron densities observed in anions and systems carrying lone electron pairs. The difference is still visible in case of the singly augmented bases (aug-cc-pVnZ, aug-mcc-pVnZ) – the energies are still too high, the corresponding entropies too low, except for the largest basis set in this series (aug-mcc-pV8Z) which converges toward the value of the best estimate (d-aug-mcc-pV8Z).

Comparison of the double– and triple–augmented version of the cc-pVnZ bases reveals a significant difference. While in case of the double augmented bases the entanglement entropies show an almost constant dependency with respect to *n*, a monotonic decrease with a notable deviation at *n* = 2 is observed for the triple–augmented basis sets. Considering that the latter are higher in quality (which is also reflected by lower energy values), it becomes evident that the entanglement entropies are not an absolute measure for the quality of the description of the system when basis sets of insufficient size are used. This conclusion is underlined by the convergence of the largest basis sets used for H^−^ (d-aug-cc-pVnZ, t-aug-cc-pVnZ, d-aug-mcc-pVnZ), yielding almost identical data for *n* = 3 and beyond.

Again the findings are in contrast to Collins' conjecture (Collins, 1993) and underline the conclusion drawn earlier in the helium case. Despite the dramatically different convergence characteristics of the entanglement entropies, a convergence toward the associated basis set limit is observed even for the unaugmented bases (cc-pVnZ, mcc-pVnZ).

Comparison of *S*_*vN*_, *S*_lin_ and *S*_occ_ reveals essentially similar plots, showing only small differences between the graphs obtained for a partiular basis set family. In case of the occupation number entropy the difference between the aug-mcc-pVnZ and d-aug-mcc-pVnZ bases is smaller compared to the other two entanglement measures.

The entropy derivatives ∂*S*/∂*Z* show the largest differences among the individual types of bases, demonstrating once more the sensitivity of this property on the chosen one-electron basis. Essentially, only the best bases (d-aug-cc-pVnZ, t-aug-cc-pVnZ, d-aug-mcc-pVnZ) show a consistent convergence, while the basis sets of lower quality show noticeable deviations in case of all employed entanglement measures.

Even worse, the unaugmented bases sets (cc-pVnZ and mcc-pVnZ) show a particularly bad behavior for *n* < 5: similar as in the helium case using the 3-21G basis, positive derivatives are observed, which corresponds again to a qualitatively wrong description of the electron entanglement. Interestingly, the same behavior is observed in case of all Pople–type basis sets considered: if no diffuse functions are applied, positive values for ∂*S*/∂*Z* are obtained (see Table S4). Despite the fact that the derivatives are negative in case large *n* or the application of diffuse functions, the data strongly suggests that at least double-augmented basis sets with *n* = 5 are required to achieve converged results in case of hydride, while the use of a single augmented basis with *n* = 6 appears to be sufficient for helium.

**Figure 3:**
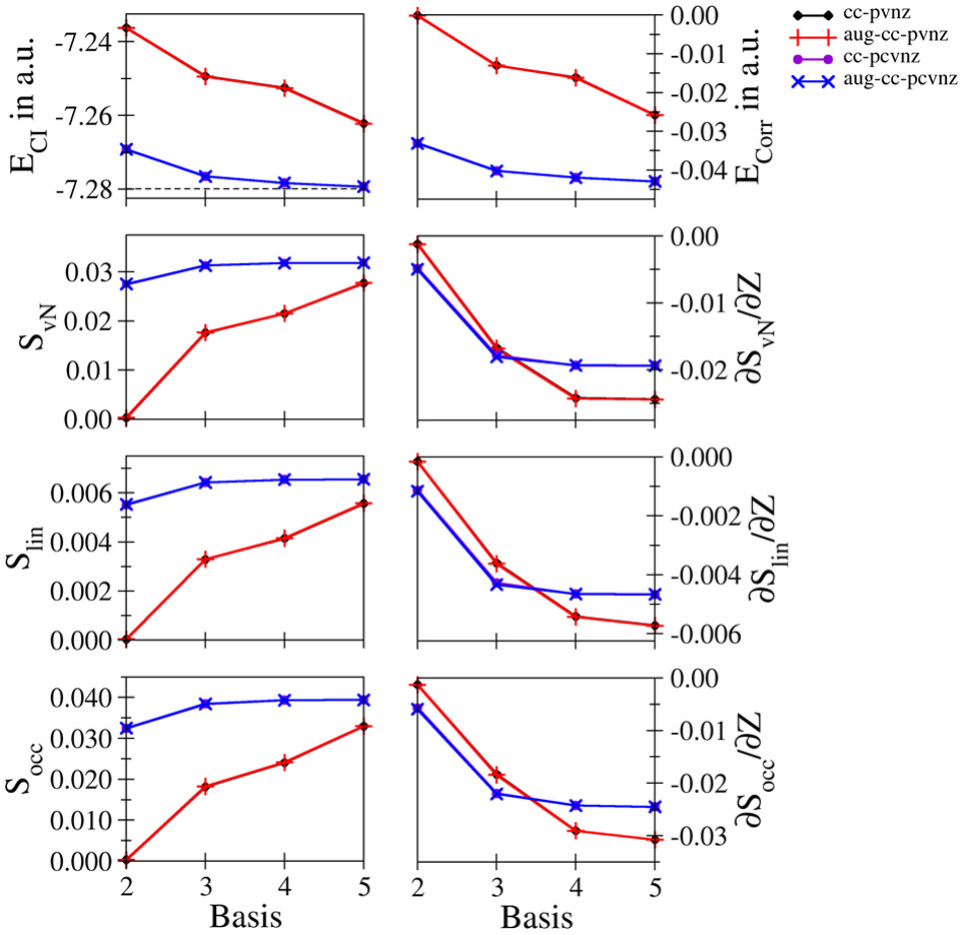
**Total and correlation energy *E*_*CI*_ and *E*_corr_ in atomic units; von Neumann *S*_*vN*_, linear *S*_lin_ and occupation number *S*_occ_ entropies and their derivative with respect to the nucleic charge *Z* obtained for Li^+^ at the FCI level using different basis sets**.

**Figure 4:**
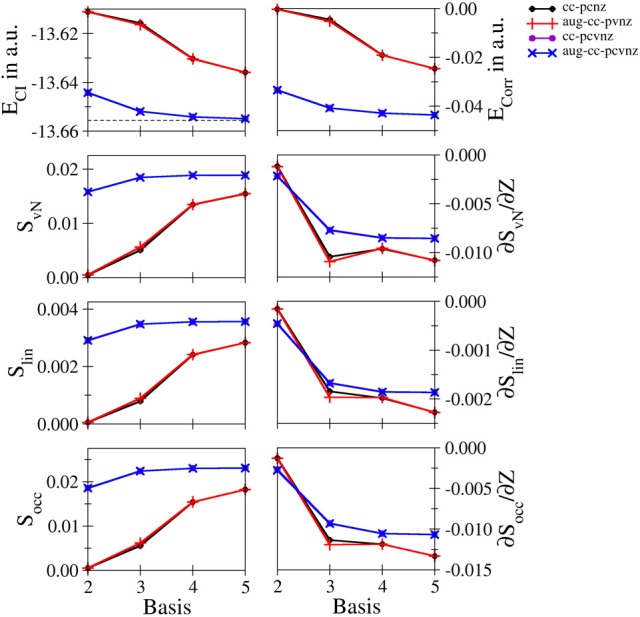
**Total and correlation energy *E*_*CI*_ and *E*_corr_ in atomic units; von Neumann *S*_*vN*_, linear *S*_lin_ and occupation number *S*_occ_ entropies and their derivative with respect to the nucleic charge *Z* obtained for Be^2+^ at the FCI level using different basis sets**.

**Li^+^ and Be^2+^**: Energetic and entropic data as well as the derivatives of the entanglement entropies are shown in Figures 3, 4 for Li^+^ and Be^2+^, the respective data are listed in Tables S6, S7. Since both systems show similar convergence properties, the discussion is focused on both systems. Due to the cationic nature of these systems and the associated contraction of the electron density, the inclusion of core functions is known to significantly improve the description of the system, which is reflected by the dramatic decrease in energy when using the cc-pCVnZ set: the energy obtained for the smallest basis in this series (cc-pCVdZ) is significantly lower than that of the largest basis of the cc-pVnZ series (*n* = 5). On the other hand, the inclusion of augmentation function does not lead to any notable improvement. This trend is also reflected by the different entanglement entropies, which again show essentially identical convergence properties. While the use of augmentation functions results only in minor changes of the entropies, a significant improvement is observed in case core functions are included.

Nevertheless, since the cc-pCVnZ series is limited to *n* = 5, the overall convergence in energy is not as accurate as observed for helium and hydride, being approximately one order of magnitude larger. The difference in energies obtained with the largest basis set (aug-cc-pCV5Z) compared to the accurate data of Manzano et al. (2010) is 5.814134 · 10^−4^ and 6.377994 · 10^−4^ for of Li^+^ and Be^2+^, respectively, being on the order of 0.5 milli–Hartree. Nevertheless, the different entanglement measures appear well converged at this level of the one–electron basis.

The derivatives of *S* with respect to *Z* show again the highest sensitivity. For both systems the graphs obtained for the cc-pVnZ bases intersect the graphs resulting from the cc-pCVnZ bases, although at different sizes of the basis sets. Although it is not possible to identify the convergence limit of the entropy derivatives, the shape of the graph resulting for the cc-pVnZ bases observed in case of Be^2+^ hints toward an oscillatory convergence. Considering also the large deviation in energy. It can be expected that the derivatives converge toward higher numbers in case basis sets with higher *n* were available for these systems. On the other hand since the values for the derivatives are considerably lower than in case of helium and hydride, the numerical differentiation given in equation (8) might not be as accurate.

**Figure 5:**
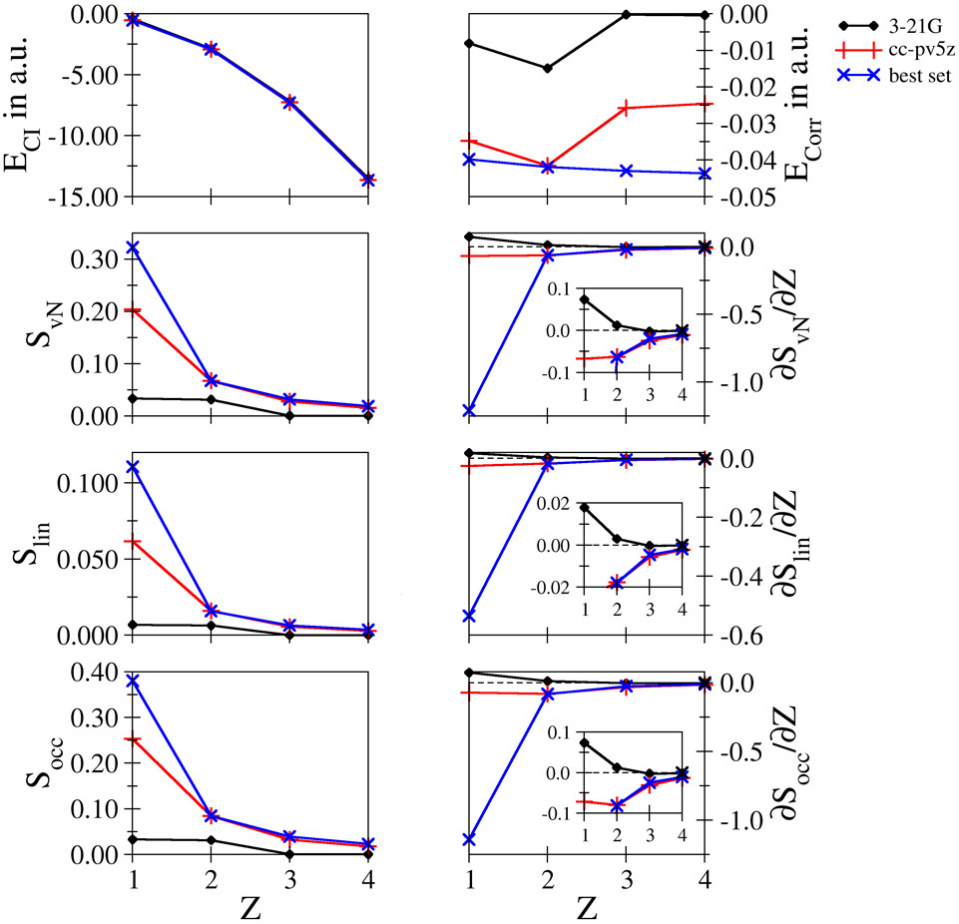
**Total and correlation energy *E*_*CI*_ and *E*_corr_ in atomic units; von Neumann *S*_*vN*_, linear *S*_lin_ and occupation number *S*_occ_ entropies and their derivative with respect to the nucleic charge *Z* obtained for H^−^, He, Li^+^ and Be^2+^ at the FCI level using the 3-21G, cc-pV5Z and the 'best' bases**.

**Comparison of the different systems**: In Figure 5 a comparison of the data obtained for the four systems is depicted for the low– and intermediate–quality bases 3-21 G and cc-pV5Z as well as the results obtained for the largest bases considered for each system being d-aug-mcc-pV8Z for hydride, t-aug-cc-pV8Z for helium and aug-cc-pCV5Z in case of Li^+^ and Be^2+^, respectively. This data is referred to as “best set” in Figure 5.

It can be seen that in case of the total energy the difference in quality between the individual data sets is not visible. This results from the fact that the energy differences between the systems are magnitudes larger than the deviations between the different basis sets. However, comparison of the respective correlation energies reveals dramatic differences. As a consequence of the increasing nucleic charge, the electron density becomes more contracted around the nucleus and correlation effects should become more prominent. This behavior is reflected by the decrease of the correlation energy upon increasing *Z* observed in case of the best set, while in case of the 3-21 G and cc-pV5Z bases a wrong trend is observed. The decrease of *E*_corr_ from hydride to helium is too pronounced resulting from the lack of diffuse functions in case of H^−^, while the absence of core functions in case of the cationic systems result in too small values for the correlation energy.

As expected the entanglement entropies prove to be sensitive to the quality of the bases, especially in the 3-21 G case, which dramatically underestimates the entanglement in line with the poor description of the correlation energy. The performance of the cc-pV5Z series is already significantly improved, however, also in this case the missing augmentation via diffuse functions leads to a large error for the hydride system, while the deviation in case of the other systems are small. Aside from small shifts of the individual values, all three entanglement measures result in very similar plots.

As became evident in the discussion of the individual systems the derivatives of the entanglement measures with respect to the nucleic charge proved to be very sensitive convergence measures. In case of the 3-21 G basis set positive derivatives are obtained in case of hydride and helium, which is in contrast to the dependence of the individual entropies, which decrease upon increasing *Z*. Clearly, the 3-21 G bases are too small to enable an adequate correlated *ab initio* treatment and should, therefore, be avoided in future studies of correlation and/or entanglement. Improving the bases to the cc-pV5Z level leads again to a large error in case of hydride, whereas the deviations for all other systems appear small on this scale. However, as discussed earlier, the use of large basis sets is highly recommended to achieve results near the basis set limit.

**Comparison of the entanglement entropies**: The linear entropy *S*_lin_ comprises a simplified version of the von Neumann entropy, obtained by expressing the logarithm via its series expression and retaining only the linear term. Thus, within a constant the two measures should yield the same result. In order to investigate the accuracy of this conjecture linear regression was applied to plots of the von Neumann entropy versus the linear entropy, the resulting correlation coefficients are listed in Table 2.

**Table 2:** **Correlation coefficients *R* resulting from linear regression of the von Neumann and linear entropies**.

***S*_*vN*_:*S*_*lin*_**	**H^-^**	**He**	**Li^+^**	**Be^2+^**
cc-pVnZ	0.9991901	0.9999984	0.9987390	0.9991970
aug-cc-pVnZ	0.9999984	0.9999999	0.9987450	0.9991194
d-aug-cc-pVnZ	0.9999997	0.9999999	–	–
t-aug-cc-pVnZ	0.9999978	0.9999999	–	–
mcc-pVnZ	0.9996481	–	–	–
aug-mcc-pVnZ	0.9999994	–	–	–
d-aug-mcc-pVnZ	0.9999995	–	–	–
cc-pCVnZ	–	–	0.9999991	0.9999987
aug-cc-pCVnZ	–	–	0.9999991	0.9999988

**Figure 6:**
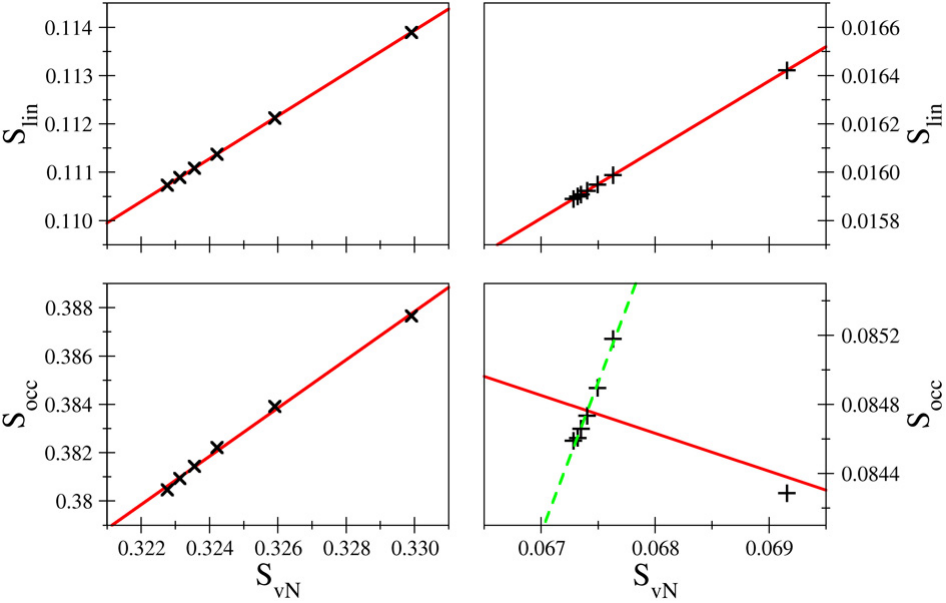
**Comparison of *S*_lin_ and *S*_occ_ as a function of *S*_*vN*_ in case of H^−^ (left) and helium (right) treated with d-aug-cc-pVnZ and t-aug-cc-pVnZ bases, respectively**. While the linear entropy correlates very well with the von Neumann entropy in all cases, the occupation number entropy does not show a linear correlation in all cases.

The correlation coefficients *R* imply an excellent linear behavior between *S*_lin_ and *S*_*vN*_, although due to the unequal spacing of data points as demonstrated in Figure 6 the correlation coefficients may appear slightly too favorable. Nevertheless, the linearity implied in *S*_lin_ is nicely fulfilled in case of all systems and bases, which is, however, not the case for the occupation number entropy (see Figure 6). While in some cases a near-linear dependence of *S*_occ_ and *S*_*vN*_ is observed as for example in the case of hydride treated with the d-aug-cc-pVnZ series (*R* = 0.998937), such an behavior is not observed in general as demonstrated also in Figure 6 for helium treated at the t-aug-cc-pVnZ level. However, if the outlier resulting from the smallest basis set in the series is not considered, the linear dependence is significantly improved (*R* = 0.993304). This finding is inline with the conclusion drawn earlier, that the agreement between *S*_occ_ and *S*_*vN*_ improves for larger basis sets.

## 4. Conclusion

The conclusion that electron correlation is indeed a physical effect related to the entanglement of electrons marks an important finding for the understanding of electronic structure theory and the systematic investigation of the convergence of entanglement measures upon increase of the one–electron basis presented in this study revealed a number of important properties of this phenomenon.

**Figure 7:**
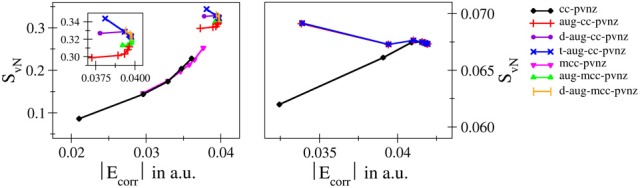
**Correlation of *S*_*vN*_ against *E*_corr_ obtained for H^−^ (left) and helium (right) using for different types of basis sets**. The varying convergence properties clearly indicate that Collins' conjecture does not hold in case of small-sized one-electron bases.

The most essential one is definitively the finding that in contrast to the total and correlation energies entanglement entropies do not show a monotonic convergence upon a systematic increase of the one–electron basis along established series such as cc-pVnZ and related bases. Monotonically increasing, monotonically decreasing as well as oscillatory convergence has been observed. These properties do not only vary for different systems, but changes in the convergence behavior have also been observed in case different bases are applied to the same system. This implies that although a relation between entanglement and electron correlation can be established based on the violation of Bell's inequality, the respective energies and the employed entanglement measures do not directly depend on each other in case too small bases are employed. This behavior demonstrated in detail in Figure 7 by plotting the von Neumann entropy against the respective correlation energy violates the conjecture given by Collins (1993) and hence, it appears that this statement should be extended, i.e., the correlation energy of a system is proportional to the respective entropy of entanglement, if a sufficiently large basis set is used ensuring data close to the basis set limit.

It should be noted at this point that in his article Collins considered electron density representations derived from experimental information such as X-ray diffraction. Although the connection between entropy and an N-representable one-particle density matrix has been discussed, errors resulting from finite–sized bases in quantum chemical computations were not within the scope of Collins' discussion. Since the electron density data obtained from experiment is not subject to basis set effects, the requirement of considering complete basis sets can be interpreted as an implicit prerequisite in Collins' conjecture. Nevertheless, it appeared desirable to explicitly highlight this point based on data obtained from actual computations.

Using the largest bases available in literature good agreement of energies and entanglement entropies with highly accurate data obtained via a Kinoshita type formulation is achieved, the deviations being on the order of about 0.05 to 0.5 milli-Hartree. However, in order to achieve consistent data the nature of the chemical system has to be taken into account when selecting the basis set. In the case of anions the use of augmentation functions to properly describe diffuse electron densities is known to be important, which is well reflected by the convergence of the individual entanglement measures. For cationic systems the inclusion of core functions proved to be crucial— the simplest basis set used in this study including core functions (cc-pCVdZ) proved to be more accurate than the largest basis without those functions (aug-cc-pV5Z) in case of Li^+^ and Be^2+^, respectively.

The use of too small one–electron bases such as the widely used 3-21 G and 6-31 G basis sets proved to be unsuitable for the characterization of entanglement phenomena aside from proof-of-concept-type calculations. In addition to the quantitatively poor entanglement entropies their derivatives with respect to the nucleic charge proved to be qualitatively wrong in case of the hydride and helium systems, resulting in ∂*S*/∂*Z* values of wrong sign. The recommendation to use basis sets explicitly designed for correlated ab initio computations is, thus, well reflected by the entanglement entropies. In general the entropy derivatives proved to be sensitive convergence measures, despite the numerical nature of the differentiation employed in this study.

Finally, regression analyses confirmed that the linear entropy *S*_lin_ shows indeed a highly linear dependence with respect to the von Neumann entropy *S*_*vN*_, whereas in the case of the occupation number entropy *S*_occ_ linearity can essentially only be observed for sufficiently large basis sets.

Although the data presented in this study is focused on the benchmarking of the entanglement measures with respect to the investigated systems and employed one–electron bases, important implications for physics and chemistry in general can be drawn from the respective findings. In particular the standard concept to describe the occupation of orbitals in chemical systems via the assignment of pairs of electrons to individual orbitals corresponds essentially to the approximate Hartree-Fock picture. However, to achieve an accurate description of systems as simple as helium, an effective occupation as observed in the NBO analysis for the FCI case given in Table 1 has to be considered. In addition to the difficulties arising from changing the established picture of single-orbital occupation used for decades in teaching and research, the formulation of a physically/chemically satisfying interpretation of such an effective occupation is a challenge of its own. The reason for this lies in the inadequate concept that the electronic structure could be described via single particle wave functions. Since this approach is an approximation, it is of course a delicate matter to map the more sophisticated full configuration interaction approach using multiple Slater determinants back onto the approximate Hartree–Fock level employing only a single determinant. Consequently, any interpretation of entanglement phenomena in a framework of single–particle occupation has to be unsatisfactory, especially since the latter can be shown to not account for these effects at all.

Despite the important finding that correlation effects have a physical counterpart, providing experimental evidence of electron–electron entanglement in real systems appears to be very a challenging task. An experiment would have to be designed in which the electrons of a test system such as helium are artificially disentangled. In case such a state could be indeed generated in an experimental setup, it can be expected that the associated energy spectrum correspond to the energy differences of the Hartree–Fock solutions obtained for various excited states and the ground state.

A related question is the difficulty to give an interpretation of the entanglement entropy and to identify the source leading to the different values observed for various systems. For example considering again the 'best set' defined earlier the ratio of the von Neumann entropies of H^−^ and Be^2+^ is 17.1. This does, however, not necessarily imply that the electrons in hydride are more entangled by a factor of 17 than those in Be^2+^. Despite the possibility to compute and analyse entanglement properties from first principles via quantum chemical computations, no information regarding the underlying mechanism can be obtained from the presented data. Aside from the chosen one–electron basis the nucleic charge is the only external parameter that influences the wave-function and thus, the entanglement/correlation of the electrons. Although disentanglement is to some extend related to the phenomenon of decoherence, it was recently discussed that these two properties are not always occurring simultaneously (Ford and O'Connell, 2010). Recently, it was shown that in addition to entanglement sudden death (ESD, also referred to as early stage disentanglement) sudden rebirth of entanglement from disentangled state can be observed as well (Yönaç et al., 2007; Yu and Eberly, 2009). Despite the fact that full configuration interaction does not provide any information regarding the time–evolution of a system, sudden death/birth of entanglement may provide a means to interpret the decreasing von Neumann entropy upon increase of the nucleic charge. Since only the electrons are included in the quantum mechanical treatment, the nuclei have to be considered as environment. The increasing ratio of the electron–nucleic potential compared to the electron–electron interaction can be interpreted as a increase of decoherence resulting in a higher probability of ESD to occur. Thus, it can be expected that periods of disentanglement increase with increasing nucleic charge.

In case it were truly possible to experimentally disentangle electrons in real atomic systems, the question arises whether it is also possible to devise an experiment setup capable of controlling (or even increasing) the degree of entanglement of the subatomic particles. While such experiments can hardly be envisaged at present, it is to be hoped that further investigations of electron-electron entanglement will improve the understanding of this phenomenon and promote further theoretical and experimental research into this fascinating field. Future investigations will, therefore, focus on the characterization of entanglement measures and their respective convergence properties in excited states as well as molecular systems such as the hydrogen molecule.

### Conflict of interest statement

The authors declare that the research was conducted in the absence of any commercial or financial relationships that could be construed as a potential conflict of interest.
