# Supplementary material for: On the basis set convergence of electron–electron entanglement measures: helium-like systems
Source: Front Chem. 2013 Nov 1;1:24. doi: 10.3389/fchem.2013.00024 (PMC3982574; doi:10.3389/fchem.2013.00024)
Supplement: Supplementary file 1 [file DataSheet1.PDF]

On the basis set convergence of electron–electron entanglement measures: helium-like systems – Supporting Information

Thomas S. Hofer

Theoretical Chemistry Division
Institute of General, Inorganic and Theoretical Chemistry
University of Innsbruck, Innrain 80-82, A-6020 Innsbruck, Austria
E-Mail: T.Hofer@uibk.ac.at
Tel.: +43-512-507-57102

September 10, 2013

The full reference for the gaussian09–programm is:

M. J. Frisch, G. W. Trucks, H. B. Schlegel, G. E. Scuseria, M. A. Robb, J. R. Cheeseman, G. Scalmani, V. Barone, B. Mennucci, G. A. Petersson, H. Nakatsuji, M. Caricato, X. Li, H. P. Hratchian, A. F. Izmaylov, J. Bloino, G. Zheng, J. L. Sonnenberg, M. Hada, M. Ehara, K. Toyota, R. Fukuda, J. Hasegawa, M. Ishida, T. Nakajima, Y. Honda, O. Kitao, H. Nakai, T. Vreven, Montgomery, Jr., J. A., J. E. Peralta, F. Ogliaro, M. Bearpark, J. J. Heyd, E. Brothers, K. N. Kudin, V. N. Staroverov, R. Kobayashi, J. Normand, K. Raghavachari, A. Rendell, J. C. Burant, S. S. Iyengar, J. Tomasi, M. Cossi, N. Rega, J. M. Millam, M. Klene, J. E. Knox, J. B. Cross, V. Bakken, C. Adamo, J. Jaramillo, R. Gomperts, R. E. Stratmann, O. Yazyev, A. J. Austin, R. Cammi, C. Pomelli, J. W. Ochterski, R. L. Martin, K. Morokuma, V. G. Zakrzewski, G. A. Voth, P. Salvador, J. J. Dannenberg, S. Dapprich, A. D. Daniels, Ö. Farkas, J. B. Foresman, J. V. Ortiz, J. Cioslowski and D. J. Fox. Gaussian 09, Gaussian Inc. Wallingford CT 2009

n	cc-pVnZ	aug-cc-pVnZ	d-aug-cc-pVnZ	t-aug-cc-pVnZ
		H^-		
2	(4s1p)/[2s1p]	(5s2p)/[3s2p]	(6s3p)/[4s3p]	(7s4p)/[5s4p]
3	(5s2p1d)/[3s2p1d]	(5s2p1d)/[4s3p2d]	(7s4p3d)/[5s4p3d]	(8s5p4d)/[6s5p4d]
4	(6s3p2d1f)/[4s3p2d1f]	(7s4p3d2f)/[5s4p3d2f]	(8s5p4d3f)/[6s5p4d3f]	(9s6p5d4f)/[7s6p5d4f]
5	(8s4p3d2f1g)/[5s4p3d2f1g]	(9s5p4d3f2g)/[6s5p4d3f2g]	(10s6p5d4f3g)/[7s6p5d4f3g]	(11s7p6d5f4g)/[8s7p6d5f4g]
6	(10s5p4d3f2g1h)/[6s5p4d3f2g1h]	(11s5p4d3f2g1h)/[7s6p5d4f3g2h]	(12s7p6d5f4g3h)/[8s7p6d5f4g3h]	(13s8p7d6f5g4h)/[9s8p7d6f5g4h]
		He		
n	cc-pVnZ	aug-cc-pVnZ	d-aug-cc-pVnZ	t-aug-cc-pVnZ
2	(4s1p)/[2s1p]	(5s2p)/[3s2p]	(6s3p)/[4s3p]	(7s4p)/[5s4p]
3	(6s2p1d)/[3s2p1d]	(7s3p2d)/[4s3p2d]	(8s4p3d)/[5s4p3d]	(9s5p4d)/[6s5p4d]
4	(7s3p2d1f)/[4s3p2d1f]	(8s4p3d2f)/[5s4p3d2f]	(9s5p4d3f)/[6s5p4d3f]	(10s6p5d4f)/[7s6p5d4f]
5	(8s4p3d2f1g)/[5s4p3d2f1g]	(9s5p4d3f2g)/[6s5p4d3f2g]	(10s6p5d4f3g)/[7s6p5d4f3g]	(11s7p6d5f4g)/[8s7p6d5f4g]
6	(10s5p4d3f2g1h)/[6s5p4d3f2g1h]	(11s6p5d4f3g2h)/[7s6p5d4f3g2h]	(12s7p6d5f4g3h)/[8s7p6d5f4g3h]	(13s8p7d6f5g4h)/[9s8p7d6f5g4h]
7	(18s6p5d4f3g2h1i)/[7s6p5d4f3g2h1i]	(19s7p6d5f4g3h2i)/[8s7p6d5f4g3h2i]	(20s8p7d6f5g4h3i)/[9s8p7d6f5g4h3i]	(21s9p8d7f6g5h4i)/[10s9p8d7f6g5h4i]
8	(21s7p6d5f4g3h2i1k)/[8s7p6d5f4g3h2i1k]	(22s8p7d6f5g4h3i2k)/[9s8p7d6f5g4h3i2k]	(23s9p8d7f6g5h4i3k)/[10s9p8d7f6g5h4i3k]	(24s10p9d8f7g6h5i4k)/[11s10p9d8f7g6h5i4k]
		Li^+		
n	cc-pVnZ	aug-cc-pVnZ	cc-pCVnZ	aug-cc-pCVnZ
2	(17s4p1d)/[3s2p1d]	(18s5p2d)/[4s3p2d]	(18s5p1d)/[4s3p1d]	(19s6p2d)/[5s4p2d]
3	(20s5p2d1f)/[4s3p2d1f]	(21s6p3d2f)/[5s4p3d2f]	(22s7p3d1f)/[6s5p3d1f]	(23s8p4d2f)/[7s6p4d2f]
4	(21s6p3d2f1g)/[5s4p3d2f1g]	(22s7p4d3f2g)/[6s5p4d3f2g]	(24s9p5d3f1g)/[8s7p5d3f1g]	(25s10p6d4f2g)/[9s8p6d4f2g]
5	(24s7p4d3f2g1h)/[6s5p4d3f2g1h]	(25s8p5d4f3g2h)/[7s6p5d4f3g2h]	(36s16p7d5f3g1h)/[10s9p7d5f3g1h]	(37s17p8d6f4g2h)/[11s10p8d6f5g2h]
		Be^{2+}		
n	cc-pVnZ	aug-cc-pVnZ	cc-pCVnZ	aug-cc-pCVnZ
2	(17s4p1d)/[3s2p1d]	(18s5p2d)/[4s3p2d]	(18s5p1d)/[4s3p1d]	(19s6p2d)/[5s4p2d]
3	(20s5p2d1f)/[4s3p2d1f]	(21s6p3d2f)/[5s4p3d2f]	(22s7p3d1f)/[6s5p3d1f]	(23s8p4d2f)/[7s6p4d2f]
4	(21s6p3d2f1g)/[5s4p3d2f1g]	(22s7p4d3f2g)/[6s5p4d3f2g]	(24s9p5d3f1g)/[8s7p5d3f1g]	(25s10p6d4f2g)/[9s8p6d4f2g]
5	(24s8p4d3f2g1h)/[6s5p4d3f2g1h]	(25s8p5d4f3g2h)/[7s6p5d4f3g2h]	(36s16p7d5f3g1h)/[10s9p7d5f3g1h]	(37s17p8d6f4g2h)/[11s10p8d6f5g2h]

Table S1: Correlation–consistent basis sets[1–6] employed for H^-, He, Li^+ and Be^{2+} given in the commonly used reference style (Gaussian primitives)/[contracted functions].

	H^-		
n	mcc-pVnZ	aug-mcc-pVnZ	d-aug-mcc-pVnZ
3	(7s2p1d)/[3s2p1d]	(8s3p2d)/[4s3p2d]	(9s4p3d)/[5s4p3d]
4	(9s3p2d1f)/[4s3p2d1f]	(10s4p3d2f)/[5s4p3d2f]	(11s5p4d3f)/[6s5p4d3f]
5	(11s4p3d2f1g)/[5s4p3d2f1g]	(12s5p4d3f2g)/[6s5p4d3f2g]	(13s6p5d4f3g)/[7s6p5d4f2g]
6	(13s5p4d3f2g1h)/[6s5p4d3f2g1h]	(14s6p5d4f3g2h)/[7s6p5d4f3g2h]	(15s7p6d5f4g3h)/[8s7p6d5f4g3h]
7	(15s6p5d4f3g2h1i)/[7s6p5d4f3g2h1i]	(16s7p6d5f4g3h2i)/[8s7p6d5f4g3h2i]	(17s8p7d6f5g4h3i)/[9s8p7d6f5g4h3i]
8	(15s7p6d5f4g3h2i1k)/[8s7p6d5f4g3h2i1k]	(16s8p7d6f5g4h3i2k)/[9s8p7d6f5g4h3i2k]	(17s9p8d7f6g5h4i3k)/[10s9p8d7f6g5h4i3k]

Table S2: Modified correlation–consistent basis sets [7–9] used for H^- given in the common used reference style (Gaussian primitives)/[contracted functions].

n	E_{CISD}	E_{corr}	S_{vN}	S_{lin}	S_{occ}	$\partial S_{vN}/\partial Z$	$\partial S_{lin}/\partial Z$	$\partial S_{occ}/\partial Z$
100[a]	-2.9037243770			$0.015914 \pm 4.4 \cdot 10^{-5}$				
				cc-pVnZ				
3-21G	-2.8505766996	-0.014896826	0.03131915	0.00642495	0.03131695	0.01243474	0.00298680	0.01259150
6-31G	-2.8701621389	-0.015001713	0.04020879	0.00861715	0.04017905	-0.01868884	-0.00472265	-0.01894119
6-311G	-2.8764183603	-0.016522936	0.03957741	0.00845786	0.03997175	-0.04000940	-0.01007710	-0.04006634
6-31G(d,p)	-2.8873650277	-0.032204602	0.06433157	0.01505828	0.07727300	-0.02452455	-0.00686995	-0.03018731
6-31+G(d,p)	-2.8880538653	-0.032403321	0.06557826	0.01540829	0.07863125	-0.04280202	-0.01204328	-0.05038510
6-311G(d,p)	-2.8905711448	-0.030675720	0.06522153	0.01530798	0.07908586	-0.06419788	-0.01804066	-0.08092666
6-311++G(d,p)	-2.8907057350	-0.030721814	0.06551824	0.01539140	0.07936017	-0.07112151	-0.02000733	-0.08833381
6-31G(2d,2p)	-2.8908546903	-0.035694264	0.06872281	0.01629795	0.08447751	-0.04450142	-0.01265882	-0.05829870
6-31++G(2d,2p)	-2.8915553638	-0.035904820	0.06994691	0.01664688	0.08586851	-0.06244450	-0.01783663	-0.07885493
6-311G(2d,2p)	-2.8957780960	-0.035882671	0.06721323	0.01586965	0.08298338	-0.06161575	-0.01743642	-0.07936077
6-311++G(2d,2p)	-2.8959201983	-0.035936277	0.06749889	0.01595052	0.08327131	-0.06825819	-0.01933526	-0.08679369
				cc-pVnZ				
2	-2.8875948311	-0.032434354	0.06198484	0.01440369	0.07402165	-0.01754201	-0.00487218	-0.02022546
3	-2.9002321690	-0.039078824	0.06611547	0.01555959	0.08243611	-0.05293601	-0.01492290	-0.06554205
4	-2.9024108779	-0.040896651	0.06745119	0.01593701	0.08475611	-0.06101365	-0.01728026	-0.07705068
5	-2.9031518775	-0.041527044	0.06748496	0.01594658	0.08488182	-0.06264291	-0.01774377	-0.08017082
6	-2.9034321188	-0.041759152	0.06743350	0.01593201	0.08478837	-0.06354386	-0.01799577	-0.08126992
7	-2.9035478542	-0.041868897	0.06737501	0.01591544	0.08470352	-0.06359944	-0.01800786	-0.08141717
8	-2.9036038589	-0.041924045	0.06733935	0.01590535	0.08463617	-0.06352562	-0.01798475	-0.08140816
				aug-cc-pVnZ				
2	-2.8895484854	-0.033843818	0.06911011	0.01640820	0.08418061	-0.0599245	-0.01706853	-0.07702607
3	-2.9005979229	-0.039414497	0.06728316	0.01588944	0.08458704	-0.0630141	-0.01783645	-0.08108260
4	-2.9025335994	-0.041011604	0.06763573	0.01598930	0.08523865	-0.0640183	-0.01814280	-0.08205173
5	-2.9032005888	-0.041573610	0.06749712	0.01595002	0.08489822	-0.0638316	-0.01808124	-0.08158308
6	-2.9034553532	-0.041782224	0.06740498	0.01592393	0.08473633	-0.0636467	-0.01802311	-0.08145009
7	-2.9035619862	-0.041882998	0.06734957	0.01590824	0.08465962	-0.0636122	-0.01800989	-0.08135735
8	-2.9036114772	-0.041931659	0.06732135	0.01590025	0.08460612	-0.0633968	-0.01794714	-0.08132302
				d-aug-cc-pVnZ				
2	-2.8895943584	-0.033884972	0.06916137	0.01642280	0.08428260	-0.0610220	-0.01738413	-0.07839969
3	-2.9006081281	-0.039424259	0.06728403	0.01588968	0.08458940	-0.0630711	-0.01785264	-0.08122998
4	-2.9025366072	-0.041014268	0.06763133	0.01598805	0.08518005	-0.0640915	-0.01816326	-0.08195057
5	-2.9032019443	-0.041574770	0.06749518	0.01594947	0.08489558	-0.0636141	-0.01801950	-0.08159907
6	-2.9034563549	-0.041782894	0.06740364	0.01592355	0.08473439	-0.0634893	-0.01797847	-0.08144591
7	-2.9035623820	-0.041883394	0.06734919	0.01590813	0.08465883	-0.0635456	-0.01799101	-0.08136443
8	-2.9036117033	-0.041931881	0.06732096	0.01590014	0.08460556	-0.0635516	-0.01799095	-0.08132175
				t-aug-cc-pVnZ				
2	-2.8896018054	-0.033890359	0.06915793	0.01642181	0.08428493	-0.06127177	-0.01745508	-0.07863770
3	-2.9006100737	-0.039426011	0.06728343	0.01588951	0.08458886	-0.06310443	-0.01786204	-0.08125172
4	-2.9025372016	-0.041014735	0.06763019	0.01598773	0.08517868	-0.06401550	-0.01814167	-0.08195837
5	-2.9032022464	-0.041574987	0.06749470	0.01594934	0.08489481	-0.06376315	-0.01806169	-0.08159993
6	-2.9034561820	-0.041782941	0.06740355	0.01592352	0.08473409	-0.06348739	-0.01797792	-0.08144654
7	-2.9035624347	-0.041883444	0.06734914	0.01590812	0.08465872	-0.06336706	-0.01794046	-0.08136415
8	-2.9036117350	-0.041931911	0.06732084	0.01590011	0.08460549	-0.06361707	-0.01800949	-0.08132134

Table S3: Total CISD energy E_{CISD} and correlation energy E_{corr} in atomic units, von Neumann, linear and occupation number entropies S_{vN}, S_{lin} and S_{occ} as well as their numerical derivatives with respect to the nuclear charge Z obtained for atomic helium employing different Pople- and Dunning–type basis sets.

[a] Kinoshita–type ansatz with N=100 [10;11]

n	E_{CISD}	E_{corr}	S_{vN}	S_{lin}	S_{occ}	$\partial S_{vN}/\partial Z$	$\partial S_{lin}/\partial Z$	$\partial S_{occ}/\partial Z$
100[a]	-0.5277510165							
t-aug-cc-pVnZ								
3-21G	-0.4085016030	-0.008080914	0.03323501	0.00688789	0.03304091	0.07373728	0.01792217	0.07385448
6-31G	-0.4313777662	-0.008935791	0.04025014	0.00862760	0.03999160	0.09548343	0.02413372	0.09571978
6-31G(d,p)	-0.4383147537	-0.015872778	0.04956281	0.01103584	0.05563600	0.10500687	0.02774432	0.11872412
6-31G(2d,2p)	-0.4478624140	-0.025420439	0.08581070	0.02129454	0.10634609	0.08286015	0.02486189	0.10309568
6-311G	-0.4816323658	-0.014960481	0.11040867	0.02892415	0.11086711	0.09314811	0.02979573	0.09641768
6-311G(d,p)	-0.4885064580	-0.021834573	0.11423348	0.03015312	0.12605980	0.10633640	0.03431986	0.13774880
6-311G(2d,2p)	-0.4961228119	-0.029450927	0.14703808	0.04112458	0.18018249	0.08105554	0.02801955	0.10573561
6-31++G(d,p)	-0.5123866303	-0.025314042	0.24888572	0.07945747	0.25256663	-0.34993559	-0.14164232	-0.34199337
6-311++G(d,p)	-0.5148719997	-0.027909261	0.25540325	0.08210658	0.26197200	-0.47891508	-0.19546640	-0.44268901
6-31++G(2d,2p)	-0.5164187500	-0.029346161	0.24022587	0.07597189	0.25939778	-0.31215004	-0.12492906	-0.26674609
6-311++G(2d,2p)	-0.5197264641	-0.032763725	0.25324869	0.08122840	0.28381679	-0.37795899	-0.15384130	-0.33112688
cc-pVnZ								
2	-0.4698567767	-0.021033051	0.08604094	0.02136365	0.09908253	0.14843488	0.04456680	0.17695925
3	-0.4963122602	-0.029620562	0.14370833	0.03997721	0.17572279	0.08152266	0.02800105	0.10831919
4	-0.5063990599	-0.032924056	0.17343949	0.05047483	0.21688399	0.01711794	0.00620384	0.02605115
5	-0.5153130617	-0.034739625	0.20386640	0.06177765	0.25373408	-0.06731219	-0.02559991	-0.07149718
6	-0.5199526399	-0.036090739	0.22769514	0.07099866	0.28232120	-0.16451172	-0.06473840	-0.18496018
aug-cc-pVnZ								
2	-0.5240286255	-0.037248349	0.29884275	0.10031158	0.36228496	-0.43932004	-0.18882001	-0.52093510
3	-0.5265621517	-0.038922559	0.30147748	0.10144566	0.36757032	-0.61131332	-0.26351994	-0.71078004
4	-0.5271392944	-0.039331180	0.30308700	0.10214011	0.36886753	-0.67703252	-0.29237370	-0.77924776
5	-0.5274289568	-0.039540147	0.30819700	0.10435309	0.37250565	-0.78274224	-0.33993930	-0.87110973
6	-0.5275452579	-0.039635608	0.31129918	0.10570264	0.37433976	-0.85658704	-0.37327473	-0.92903826
d-aug-cc-pVnZ								
2	-0.5246233133	-0.037777461	0.32682913	0.11252707	0.38399612	-1.15129707	-0.51011612	-0.98646401
3	-0.5270144194	-0.039334236	0.32863480	0.11332788	0.38606046	-1.24408733	-0.55227608	-1.15349980
4	-0.5274626304	-0.039626594	0.32498526	0.11171088	0.38274879	-1.22528809	-0.54184577	-1.14312576
5	-0.5276181309	-0.039717082	0.32361292	0.11110445	0.38137216	-1.21873657	-0.53816651	-1.14013469
6	-0.5276749936	-0.039757534	0.32300384	0.11083557	0.38068327	-1.21369818	-0.53559548	-1.13879474
t-aug-cc-pVnZ								
2	-0.5249895841	-0.038100831	0.34375386	0.12009240	0.39707176	-1.29532896	-0.58405340	-1.17635864
3	-0.5270155068	-0.039334744	0.32855922	0.11329433	0.38605049	-1.24396380	-0.55217723	-1.15332979
4	-0.5274636136	-0.039626831	0.32496459	0.11170174	0.38275063	-1.22621487	-0.54224357	-1.14373267
5	-0.5276191025	-0.039717416	0.32362260	0.11110872	0.38137779	-1.21742933	-0.53759480	-1.14085817
6	-0.5276758159	-0.039757951	0.32302262	0.11084386	0.38068990	-1.21528086	-0.53630448	-1.13944859

Table S4: Total CISD energy E_{CISD} and correlation energy E_{corr} in atomic units, von Neumann, linear and occupation number Entropies S_{vN}, S_{lin} and S_{occ} as well as their numerical derivatives with respect to the nuclear charge Z obtained for hydride employing different Pople- and Dunning–type basis sets.
[a] Manzano *et al.*[10] using a Kinoshita–type ansatz with N=100

n	E_{CISD}	E_{corr}	S_{vN}	S_{lin}	S_{occ}	$\partial S_{vN}/\partial Z$	$\partial S_{lin}/\partial Z$	$\partial S_{occ}/\partial Z$
100[a]	-0.5277510165							
				mcc-pVnZ				
3	-0.4977716107	-0.029611410	0.14621900	0.04084166	0.17810544	0.08233902	0.02841871	0.11011058
4	-0.5073147730	-0.032942110	0.17561208	0.05126365	0.21896284	0.01570197	0.00571143	0.02664571
5	-0.5137668614	-0.034606029	0.19752968	0.05937915	0.24730843	-0.04462211	-0.01680881	-0.05059225
6	-0.5171319792	-0.035764997	0.21323023	0.06536359	0.26838297	-0.10488958	-0.04044330	-0.13027416
7	-0.5195196057	-0.036462339	0.22511024	0.06998327	0.28327320	-0.15411999	-0.06043295	-0.19294001
8	-0.5234353389	-0.037648422	0.25299315	0.08112441	0.31533247	-0.29904485	-0.12168101	-0.36508752
				aug-mcc-pVnZ				
3	-0.5267269828	-0.039208775	0.31295285	0.10642391	0.37756190	-0.73171714	-0.31943450	-0.82479074
4	-0.5272712113	-0.039558693	0.31299299	0.10644143	0.37722354	-0.79626304	-0.34762770	-0.88877205
5	-0.5274894672	-0.039682639	0.31467528	0.10717655	0.37785738	-0.86791323	-0.37960009	-0.94628127
6	-0.5275778803	-0.039742272	0.31579201	0.10766527	0.37820937	-0.90971619	-0.39836398	-0.97898674
7	-0.5276300320	-0.039767695	0.31673478	0.10807832	0.37840915	-0.94724457	-0.41521933	-1.00478125
8	-0.5276832399	-0.039784037	0.31915703	0.10914148	0.37930418	-1.03477168	-0.45476773	-1.05895928
				d-aug-mcc-pVnZ				
3	-0.5269099728	-0.039390369	0.32990675	0.11389291	0.38765808	-1.24620046	-0.55395091	-1.15141188
4	-0.5273804912	-0.039666010	0.32591323	0.11212145	0.38390782	-1.22679829	-0.54304531	-1.14335941
5	-0.5275547148	-0.039745002	0.32421991	0.11137257	0.38220650	-1.21725540	-0.53785768	-1.14096392
6	-0.5276234808	-0.039784482	0.32356159	0.11108178	0.38143523	-1.21464497	-0.53633041	-1.13997732
7	-0.5276633807	-0.039797770	0.32313259	0.11089240	0.38092357	-1.21218605	-0.53500118	-1.13957683
8	-0.5276994364	-0.039797569	0.32276573	0.11073051	0.38046331	-1.21054743	-0.53407001	-1.13935676

Table S5: Total CISD energy E_{CISD} and correlation energy E_{corr} in atomic units, von Neumann, linear and occupation number Entropies S_{vN}, S_{lin} and S_{occ} as well as their numerical derivatives with respect to the nuclear charge Z obtained for hydride using different mcc-pVnZ basis sets (continued.
[a] Manzano *et al.*[10] using a Kinoshita–type ansatz with N=100

n	E_{CISD}	E_{corr}	S_{vN}	S_{lin}	S_{occ}	$\partial S_{vN}/\partial Z$	$\partial S_{lin}/\partial Z$	$\partial S_{occ}/\partial Z$
100[a]	-7.2799134127							
cc-pVnZ								
2	-7.2362237458	-0.000105104	0.00030784	0.00003577	0.00033455	-0.00120199	-0.00015243	-0.00128036
3	-7.2493533691	-0.012973298	0.01762624	0.00329375	0.01818022	-0.01669479	-0.00360116	-0.01831283
4	-7.2524931042	-0.016108747	0.02151503	0.00414826	0.02407683	-0.02419536	-0.00541014	-0.02903794
5	-7.2621768502	-0.025766149	0.02772121	0.00557079	0.03297388	-0.02438459	-0.00571907	-0.03074619
cc-pCVnZ								
2	-7.2691917361	-0.033070912	0.02749042	0.00551671	0.03245272	-0.00485696	-0.00113730	-0.00575040
3	-7.2765596159	-0.040179426	0.03131466	0.00642387	0.03841611	-0.01780632	-0.00427693	-0.02197733
4	-7.2783309478	-0.041946397	0.03180225	0.00654117	0.03933253	-0.01932190	-0.00465519	-0.02424237
5	-7.2793317742	-0.042920678	0.03184953	0.00655256	0.03941564	-0.01936571	-0.00466712	-0.02449306
aug-cc-pVnZ								
2	-7.2362306090	-0.000109564	0.00031908	0.00003719	0.00034756	-0.00122042	-0.00015532	-0.00130292
3	-7.2494192548	-0.013038866	0.01765789	0.00330058	0.01824418	-0.01678645	-0.00362209	-0.01839821
4	-7.2525754714	-0.016191010	0.02156584	0.00415963	0.02415137	-0.02426348	-0.00542772	-0.02903877
5	-7.2622350380	-0.025824323	0.02775464	0.00557863	0.03301944	-0.02439925	-0.00572384	-0.03072742
aug-cc-pCVnZ								
2	-7.2692110642	-0.033088073	0.02754055	0.00552845	0.03251143	-0.00496817	-0.00116375	-0.00594627
3	-7.2765641075	-0.040183504	0.03132374	0.00642605	0.03842775	-0.01803745	-0.00433269	-0.02202355
4	-7.2783318906	-0.041947176	0.03180295	0.00654133	0.03933359	-0.01930175	-0.00465035	-0.02424455
5	-7.2793319993	-0.042920885	0.03184967	0.00655259	0.03941592	-0.01933966	-0.00466085	-0.02449463

Table S6: Total CISD energy E_{CISD} and correlation energy E_{corr} in atomic units, von Neumann, linear and occupation number Entropies S_{vN}, S_{lin} and S_{occ} as well as their numerical derivatives with respect to the nucelar charge Z obtained for Li^{+} using different basis sets.
[a] Manzano *et al.*[10] using a Kinoshita–type ansatz with N=100

n	E_{CISD}	E_{corr}	S_{vN}	S_{lin}	S_{occ}	$\partial S_{vN}/\partial Z$	$\partial S_{lin}/\partial Z$	$\partial S_{occ}/\partial Z$
100[a]	-13.6555662384							
cc-pVnZ								
2	-13.6110962280	-0.000301629	0.00046029	0.00005552	0.00050739	-0.00116108	-0.00015341	-0.00126177
3	-13.6156821360	-0.004523711	0.00505547	0.00079321	0.00553744	-0.01043777	-0.00184611	-0.01135602
4	-13.6303549510	-0.019103639	0.01345351	0.00241358	0.01539613	-0.00962524	-0.00198160	-0.01185286
5	-13.6358920700	-0.024600729	0.01544836	0.00282925	0.01819551	-0.01078384	-0.00227312	-0.01332623
cc-pCVnZ								
2	-13.6442192980	-0.033408828	0.01580053	0.00290363	0.01852733	-0.00216650	-0.00045846	-0.00267831
3	-13.6519480340	-0.040787084	0.01847376	0.00347734	0.02236069	-0.00768898	-0.00167246	-0.00929487
4	-13.6541511680	-0.042899639	0.01884267	0.00355773	0.02302013	-0.00849834	-0.00185506	-0.01054744
5	-13.6549282860	-0.043635487	0.01886439	0.00356247	0.02306650	-0.00854558	-0.00186576	-0.01067810
aug-cc-pVnZ								
2	-13.6111159760	-0.000315597	0.00047889	0.00005799	0.00052851	-0.00119309	-0.00015829	-0.00129894
3	-13.6163721080	-0.005213608	0.00562961	0.00089556	0.00613675	-0.01094190	-0.00196556	-0.01191450
4	-13.6304040040	-0.019152441	0.01346136	0.00241520	0.01540950	-0.00956473	-0.00196934	-0.01185708
5	-13.6359212910	-0.024629932	0.01545890	0.00283147	0.01820902	-0.01081226	-0.00227938	-0.01332535
aug-cc-pCVnZ								
2	-13.6442444880	-0.033428500	0.01583465	0.00291085	0.01856797	-0.00215590	-0.00045639	-0.00277517
3	-13.6519521600	-0.040790567	0.01847821	0.00347831	0.02236598	-0.00771242	-0.00167763	-0.00931873
4	-13.6541519630	-0.042900169	0.01884288	0.00355777	0.02302052	-0.00850213	-0.00185589	-0.01054897
5	-13.6549284390	-0.043635616	0.01886441	0.00356247	0.02306655	-0.00854170	-0.00186491	-0.01067825

Table S7: Total CISD energy E_{CISD} and correlation energy E_{corr} in atomic units, von Neumann, linear and occupation number Entropies S_{vN}, S_{lin} and S_{occ} as well as their numerical derivatives with respect to the nucelar charge Z obtained for the Be^{2+} ion using different basis sets.
[a] Manzano *et al.*[10] using a Kinoshita–type ansatz with N=100

References

[1] T. H. Dunning Jr. Gaussian basis sets for use in correlated molecular calculations. I. The atoms boron through neon and hydrogen. *J. Chem. Phys.*, 90:1007–1023, 1989.

[2] R. A. Kendall, T. H. Dunning Jr., and R. J. Harrison. Electron affinities of the first-row atoms revisited. systematic basis sets and wave functions. *J. Chem. Phys.*, 96: 6796–6806, 1992.

[3] D. E. Woon and T. H. Dunning Jr. Gaussian basis sets for use in correlated molecular calculations. IV. Calculation of static electrical response properties. *J. Chem. Phys.*, 100:2975, 1994.

[4] K. A. Peterson, D. E. Woon, and T. H. Dunning Jr. Benchmark calculations with correlated molecular wave functions. IV. The classical barrier height of the $H+H_2 \to H_2+H$ reaction. *J. Chem. Phys.*, 100:7410–7415, 1994.

[5] T. van Mourik, A. K. Wilson, and T. H. Dunning, Jr. Benchmark calculations with correlated molecular wavefunctions. XIII. Potential energy curves for He_2, Ne_2, and Ar_2 using correlation consistent basis sets through augmented sextuple zeta. *Mol. Phys.*, 96:529, 1999.

[6] B. P Prascher, D. W. Woon, K. A. Peterson, T. H. Dunning, Jr, and A. K. Wilson. Gaussian basis sets for use in correlated molecular calculations. VII. Valence, core-valence, and scalar relativistic basis sets for Li, Be, Na, and Mg. *Theor. Chem. Acc.*, 40:69, 2011.

[7] S. L. Mielke, B. C. Garrett, and K. A. Peterson. The utility of many-body decompositions for the accurate basis set extrapolation of ab initio data. *J. Chem. Phys.*, 111: 3806, 1999.

[8] S. L. Mielke, B. C. Garrett, and K. A. Peterson. A hierarchical family of global analytic Born–Oppenheimer potential energy surfaces for the $H+H_2$ reaction ranging in quality from double-zeta to the complete basis set limit. *J. Chem. Phys.*, 116:4142, 2002.

[9] S. L. Mielke, D. W. Schwenke, and K. A. Peterson. Benchmark calculations of the complete configuration-interaction limit of Born–Oppenheimer diagonal corrections to the saddle points of isotopomers of the $H+H_2$ reaction. *J. Chem. Phys.*, 122:224313, 2005.

[10] D. Manzano, A. R. Plastino, J. S. Dehesa, and T. Koga. Quantum entanglement in two-electron atomic models. *J. Phys. A: Math. Theor.*, 43:275301, 2010.

[11] J. S. Dehesa, T. Koga, R. J. Yáñez, A. R. Plastino, and R. O. Esquivel. Quantum entanglement in helium. *J. Phys. B: Opt. Phys.*, 45:015504, 2012.
